# Alzheimer’s Biomarkers From Multiple Modalities Selectively Discriminate Clinical Status: Relative Importance of Salivary Metabolomics Panels, Genetic, Lifestyle, Cognitive, Functional Health and Demographic Risk Markers

**DOI:** 10.3389/fnagi.2018.00296

**Published:** 2018-10-02

**Authors:** Shraddha Sapkota, Tao Huan, Tran Tran, Jiamin Zheng, Richard Camicioli, Liang Li, Roger A. Dixon

**Affiliations:** ^1^Neuroscience and Mental Health Institute, University of Alberta, Edmonton, AB, Canada; ^2^Department of Chemistry, University of Alberta, Edmonton, AB, Canada; ^3^Department of Medicine (Neurology), University of Alberta, Edmonton, AB, Canada; ^4^Department of Psychology, University of Alberta, Edmonton, AB, Canada

**Keywords:** Alzheimer’s disease, mild cognitive impairment, cognitively normal, salivary metabolomics, biomarkers, genetics, cognition, victoria longitudinal study

## Abstract

**Background**: Among the neurodegenerative diseases of aging, sporadic Alzheimer’s disease (AD) is the most prevalent and perhaps the most feared. With virtually no success at finding pharmaceutical therapeutics for altering progressive AD after diagnosis, research attention is increasingly directed at discovering biological and other markers that detect AD risk in the long asymptomatic phase. Both early detection and precision preclinical intervention require systematic investigation of multiple modalities and combinations of AD-related biomarkers and risk factors. We extend recent unbiased metabolomics research that produced a set of metabolite biomarker panels tailored to the discrimination of cognitively normal (CN), cognitively impaired and AD patients. Specifically, we compare the prediction importance of these panels with five other sets of modifiable and non-modifiable AD risk factors (genetic, lifestyle, cognitive, functional health and bio-demographic) in three clinical groups.

**Method**: The three groups were: CN (*n* = 35), mild cognitive impairment (MCI; *n* = 25), and AD (*n* = 22). In a series of three pairwise comparisons, we used machine learning technology random forest analysis (RFA) to test relative predictive importance of up to 19 risk biomarkers from the six AD risk domains.

**Results**: The three RFA multimodal prediction analyses produced significant discriminating risk factors. First, discriminating AD from CN was the AD metabolite panel and two cognitive markers. Second, discriminating AD from MCI was the AD/MCI metabolite panel and two cognitive markers. Third, discriminating MCI from CN was the MCI metabolite panel and seven markers from four other risk modalities: genetic, lifestyle, cognition and functional health.

**Conclusions**: Salivary metabolomics biomarker panels, supplemented by other risk markers, were robust predictors of: (1) clinical differences in impairment and dementia and even; (2) subtle differences between CN and MCI. For the latter, the metabolite panel was supplemented by biomarkers that were both modifiable (e.g., functional) and non-modifiable (e.g., genetic). Comparing, integrating and identifying important multi-modal predictors may lead to novel combinations of complex risk profiles potentially indicative of neuropathological changes in asymptomatic or preclinical AD.

## Introduction

Epidemiological projections point in the direction of increased worldwide prevalence and growing burden of neurodegenerative disease, especially Alzheimer’s disease (AD; Prince et al., 2015; Alzheimer’s Association, 2016; Wimo et al., 2017). Given the lack of success in developing therapeutics to reverse the course of neurodegeneration in aging after diagnosis (Cummings et al., 2014), research and clinical attention has shifted to multimodal risk detection in asymptomatic phases (Sperling et al., 2011) so as to promote early risk management or prevention (Anstey et al., 2015). Early detection of sporadic AD may require systematic attention to multiple modalities of biomarkers and risk factors, perhaps beyond (but including) the established neurobiological and clinical hallmarks of the disease (e.g., beta amyloid; Barnes and Yaffe, 2011). Accordingly, recent research has focused on testing panels, dosages, and interactions of multiple biomarkers, examining their synergistic, modifying, or complementary influences on phenotypes, pre-clinical trajectories or clinical status (Edwards et al., 2015; McFall et al., 2015a; Iturria-Medina et al., 2016; Sapkota et al., 2017; Sapkota and Dixon, 2018). Arguably, identifying perturbations in profiles of biomarkers in asymptomatic periods of impairment or AD may provide a promising opportunity for developing precision or programmatic interventions that could delay or prevent clinical diagnosis (Imtiaz et al., 2014; Hampel et al., 2017). However, translational progress may be optimized when one or more of three conditions are available: (1) a roster of established multi-modal modifiable risk biomarkers are included; (2) these risk biomarkers can be estimated with valid but relatively non-invasive technology; and (3) comparative prediction and discrimination data are available (Anstey et al., 2015; Olanrewaju et al., 2015; Casanova et al., 2016).

We adopt a multi-modal comparative approach to determining the relative importance of multiple established risk biomarkers of cognitive impairment and AD. The six AD risk biomarker clusters include: (1) novel metabolomics biomarker panels; (2) selected AD genetic risk polymorphisms (e.g., *Apolipoprotein E* (*APOE*)); (3) functional health (e.g., vascular); (4) lifestyle engagement (e.g., physical activity); (5) cognitive performance (e.g., memory); and (6) bio-demographic factors (e.g., sex). A total of 19 risk biomarkers are available for testing simultaneously in three pairwise competitive analyses conducted with machine learning technology random forest analyses (RFA). This approach identifies the predictors that contribute most significantly to the discrimination of the clinical groups. In the present study, these groups include the benchmark cognitively normal (CN) as well as mild cognitive impairment (MCI) and AD groups. The predictors vary in the extent to which they are likely to be modifiable, an important consideration for potential downstream intervention (Barnes and Yaffe, 2011; Anstey et al., 2013b, 2015; Norton et al., 2014; Livingston et al., 2017). We limited our predictors to those that are likely to require relatively non-invasive assessment techniques. Accordingly, in the present study, both metabolomics and genetic markers were developed from salivary samples. A central aim of this study was to examine the extent to which newly discovered metabolomics biomarker panels would emerge as important predictors of MCI and AD in the competitive context of a broad range of other established and relatively non-invasive AD risk factors.

Two of the present biomarker clusters are derived from salivary samples collected in the context of longitudinal study of aging. Saliva is of interest in research on biomarkers of neurodegenerative diseases and aging for several reasons. It is a premier non-invasive biofluid, easily collected and stored (Wong, 2006) and increasingly acknowledged for its potential as a source fluid for genomic, metabolomics and candidate biomarker studies (Wishart et al., 2013; Liang et al., 2015). Its viability for DNA extraction and genotyping is well established and effectively applied in genetics of aging and dementia (McFall et al., 2016; Sapkota et al., 2017). Recently, metabolomics technology has advanced such that salivary samples have provided source fluids for biomarker discovery in AD (Liang et al., 2015; Figueira et al., 2016). In our previous work, we have used salivary samples for genotyping, biomarker network and interaction analyses (McFall et al., 2016; Sapkota et al., 2017), and metabolomics-based discovery of biomarkers of impairment and AD (Zheng et al., 2012; Huan et al., 2018). Although not yet comprehensively compared across biofluid modalities, salivary biomarkers may enable better accessibility to a wider range of worldwide and diversity samples than as yet available via more traditional biofluids (blood, cerebral spinal fluid; Hu et al., 2010; Thambisetty and Lovestone, 2010; Mousavi et al., 2014; Trushina and Mielke, 2014; Liang et al., 2016; Simpson et al., 2016; Toledo et al., 2017).

The first set of biomarkers was developed in a previous salivary metabolomics analysis of CN, MCI and AD samples. Metabolomics is a global approach to detecting perturbations in metabolic pathways that can reflect early and subtle disease-related changes in the central nervous system (Kaddurah-Daouk and Krishnan, 2009). It evaluates the metabolic state of the organism. The metabolome represents the end and transitional products of interactions between genes, proteins, and the environment (Xia et al., 2013; Jové et al., 2014; González-Domínguez et al., 2015). The result of a metabolomics analysis is an empirically and quantitatively derived set of metabolites that discriminate between two clinical groups and provide targets for further analyses of mechanisms, associations and clinical applications (Mishur and Rea, 2012; Ibáñez et al., 2013; Enche Ady et al., 2017). In the present study, we assemble a set of discovered and verified discriminant metabolite panels (comprised of more than one biomarker) from a recent salivary AD metabolomics study (Huan et al., 2018). Specifically, we developed putatively identified metabolite panels discriminating CN, MCI and AD groups (Huan et al., 2018). Unique metabolite biomarker panels were developed for each pairwise comparison and all panels displayed very high sensitivity for the comparisons. The AD biomarker panel (discriminating AD from CN) was comprised of three metabolites (Methylguanosine, Histidinyl-Phenylalanine, Choline-cytidine) that were associated with the phenylalanine and histamine biosynthesis pathways (Huan et al., 2018). The AD/MCI panel was comprised of three metabolites (Amino-dihydroxybenzene, Glucosylgalactosyl hydroxylysine − H_2_O, Aminobutyric acid + H_2_) that were provisionally associated with lipid metabolism pathways^1^. The MCI/CN panel was comprised of two metabolites (Glucosylgalactosyl hydroxylysine − H_2_O, Glutamine-carnitine) and provisionally associated with carnitine synthesis, oxidation of branched chain fatty acid, lipid and fatty acid metabolism pathways^2^. Details of the metabolomics procedures used in the earlier study are available elsewhere (Zheng et al., 2012; Huan et al., 2018) and are summarized in the present “Materials and Methods” section.

The genetic biomarker modality was also derived from salivary samples. We selected AD genetic risk markers from an available pool with relatively known properties and application in multi-modal biomarker research (Williams et al., 2010; Karch et al., 2014; Karch and Goate, 2015; Huynh and Mohan, 2017). All four were detected in genome-wide association studies and frequently linked to AD and cognitive decline (Harold et al., 2009; Lambert et al., 2009; Chibnik et al., 2011). The four genetic markers are: *APOE* rs7412, (rs429358; Brainerd et al., 2011; Dixon et al., 2014; Runge et al., 2014; Mahoney-Sanchez et al., 2016), *Complement receptor 1* (*CR1*; rs6656401; Crehan et al., 2012; Fonseca et al., 2016), *Clusterin* (*CLU*; rs11136000; Thambisetty et al., 2013; McFall et al., 2016) and *Phosphatidylinositol-binding clathrin assembly protein* (*PICALM*; rs3851179; Barral et al., 2012; Xiao et al., 2012; Ferencz et al., 2014; Morgen et al., 2014). *APOE* is the most established genetic risk factor for AD and is involved in lipid transport and metabolism (Liu et al., 2013). *CR1* may be involved in rate of Aβ_42_ clearance in AD (Lambert et al., 2009). *CLU* has been associated with regulation of lipid transport, Aβ clearance, and brain atrophy (Karch and Goate, 2015). *PICALM* is involved in Aβ peptide production and connected to Aβ metabolism and plaque formation (Xiao et al., 2012).

The remaining sets of AD risk factors have been examined in observational research, reported in reviews, and linked to early AD detection and potential prevention (Livingston et al., 2017). The functional health predictor domain included three dementia-related biomarkers: pulse pressure (PP), body mass index (BMI) and gait timed walk; Qiu et al., 2003; Dahl et al., 2013; Mielke et al., 2013; Emmerzaal et al., 2015; McDade et al., 2016; McFall et al., 2016; MacDonald et al., 2017). PP, a reliable proxy of arterial stiffness has been considered a better predictor of poor vascular health compared to systolic blood pressure alone (Raz et al., 2011; Nation et al., 2013) and linked to (1) AD biomarkers in CN and AD risk (Nation et al., 2013; McFall et al., 2016); (2) MCI (Yaneva-Sirakova et al., 2012); (3) cerebral small vessel disease (Singer et al., 2014); and (4) cognitive decline (McFall et al., 2015b). Lower late-life BMI and higher mid-life BMI has consistently been linked to increased dementia risk (Emmerzaal et al., 2015). Potential mechanisms (Emmerzaal et al., 2015) include: (1) greater inflammation (Yaffe et al., 2004); (2) structural brain changes (Pannacciulli et al., 2006); and (3) higher cholesterol levels in mid-life and lower levels in late-life (Mielke et al., 2005). The lifestyle activity predictor domain included four markers of everyday engagement, with higher levels often associated with AD risk reduction and lower levels with risk elevation. A standard self-report instrument represented levels of everyday integrative cognitive, novel cognitive, physical and social activities (Deary et al., 2006; Bherer et al., 2013; Wang et al., 2013; Vemuri et al., 2014; Thibeau et al., 2017). Cognitively stimulating lifestyle activities (Vemuri et al., 2012) and physical activities (Chen et al., 2016; Falck et al., 2017) have been shown to delay AD onset. Specifically, physical activities may lead to improvements in neurogenesis as a result of increased cerebral blood flow in the dentate gyrus (Chen et al., 2016). The cognitive performance predictor domain included four measures: episodic memory (as early cognitive manifestation associated with hippocampal dysfunction), EF (Stroop, which tests the ability to inhibit cognitive interferences; Scarpina and Tagini, 2017), speed (simple reaction time, the level of which reflects slower or faster processing speed potential indicator of early normal or preclinical cognitive decline (McFall et al., 2015a), and global cognition (assessed with the Mini-Mental State Exam (MMSE)). The bio-demographic domain included age, sex and education (Li and Singh, 2014; Schneeweis et al., 2014; Jack et al., 2015; Cadar et al., 2016; Riedel et al., 2016; Sachdev et al., 2016). Age is the most important non-modifiable risk factor for developing AD with large number of sporadic AD cases occurring after 65 years (Guerreiro and Bras, 2015). Sex differences have been observed in AD with significantly higher prevalence in women than men (Mazure and Swendsen, 2016). Lifestyle experiences and choices (i.e., diet, exercise) vary by sex and may have an indirect influence on the brain over the lifespan (Mazure and Swendsen, 2016). Education has widely been used as a proxy for cognitive reserve (Tucker and Stern, 2011; Stern, 2012, 2017). Adults with higher cognitive reserve (higher education levels) may have greater tolerance to AD pathology than those with lower cognitive reserve (lower education levels; Stern, 2012).

For each of three comparative RFA prediction models we included up to 19 predictors. RFA is a machine-learning-based data exploration technique that combines large numbers of regression tree predictions from a random sample of participants and variables (Strobl et al., 2009; McDermott et al., 2017). It accommodates multiple predictors and smaller sample sizes, producing a solution that features a rank ordering of the top important predictors of the target clinical condition. The general objective was to examine and compare the extent to which new salivary metabolite biomarker panels fared in the competitive context of other AD biomarkers in predicting clinical status in pairwise comparisons across three groups: CN, MCI and AD.

## Materials and Methods

### Participants

Participants were community-dwelling older adult volunteers from the Victoria Longitudinal Study (VLS), an ongoing multi-cohort investigation of biomedical, genetic, metabolic, functional, neurocognitive and other aspects of aging, impairment and dementia. This study was carried out in accordance with the recommendations of the Human Research Ethics Guidelines, University of Alberta with written informed consent from all subjects. All subjects gave written informed consent in accordance with the Declaration of Helsinki. The protocol was approved by the Human Research Ethics Board. Detailed information on overall VLS recruitment, research design, and participant characteristics are available elsewhere (Dixon and de Frias, 2004; McFall et al., 2015a). For the present study, the CN and MCI participants were drawn from a subset of the main cohorts that participated in the VLS biofluid and genetics initiative (2009–2012). The AD patients were recruited from the Geriatric and Cognitive Clinic at the Glenrose Rehabilitation Hospital (Edmonton). All participants (*N* = 82) received a small honorarium for their contributions. The present research includes adults classified as CN (*n* = 35; age 64–75 years; 62.9% female), MCI (*n* = 25; age 64–75 years; 60% female), and diagnosed AD (*n* = 22; age 52–91; 72.7% female). Participant demographic characteristics are presented in Table 1.

**Table 1:** Clinical characteristics of CN, MCI and AD groups^a^.

Characteristics	CN	MCI	AD
*N* (total = 82)	35	25	22
Age (years)^b^	69.94 (3.80)	70.40 (3.38)	77.09 (11.20)
Gender (M/F)	13/22	10/15	6/16
Education, years^b^	15.69 (2.69)	14.68 (2.94)	11.59 (3.23)
Mini-Mental State Exam^b^	28.46 (1.42)	27.39 (3.14)	21.32 (4.76)

*CN, Cognitively Normal; MCI, Mild Cognitive Impairment; AD, Alzheimer’s disease. ^a^Exclusionary, diagnostic, and classification criteria applied. ^b^Values are mean (standard deviation)*.

### Classification and Diagnosis

To select CN and MCI participants, we initially applied exclusionary criteria (no diagnosed dementia, cardiovascular disease, stroke history, or psychiatric illness, MMSE ≥ 24) and inclusionary criteria (two waves (4.5 years) of longitudinal data, complete data on a separate cognitive reference battery). We implemented an established and objective four-step cognitive classification procedure that requires strict adherence to specific assessment and selection rules (Dixon et al., 2007, 2014; de Frias et al., 2009; Dolcos et al., 2012; Huan et al., 2018). We conducted the full classification procedure at each of two waves (about 4.5 years apart). At both waves, eligible participants completed a five-domain cognitive battery, including measures of key domains: perceptual speed, inductive reasoning, episodic memory, verbal fluency and semantic memory. The procedure was as follows. Source participants were: (1) stratified into two age (64–73 and 74–95) and education (0–12 years and 13 + years) groups; (2) placed in appropriate age x education subgroups; (3) analyzed for mean cognitive scores on all tests; and (4) evaluated by score within respective age x education subgroups. We applied a moderate criterion to establish higher or lower (“impaired”) group based on one standard deviation below the subgroup mean for any cognitive test. For participants to be classified as CN or MCI they were required to be objectively stable in their classification at both waves (at least 4.5 years). The procedure resulted in *n* = 25 MCI participants, who we then matched (age, sex) with CN adults and supplemented with randomly selected additional participants (*n* = 35). Overall, this approach emphasizes objective and stable classification, reducing the risk of false assignments and enhancing homogeneity of the groups (Dolcos et al., 2012; Bondi et al., 2014; Dixon et al., 2014). AD patients were recruited from the Geriatric and Cognitive Neurology clinics at the Glenrose Hospital in Edmonton, Alberta. The clinical diagnosis of AD was based on the Diagnostic and Statistical Manual of Mental Disorders (4th Edition) criteria for Dementia of the Alzheimer Type. Clinical assessments were performed as part of routine clinical evaluation, which included caregiver report of cognitive decline and impaired functional status, mental status evaluation of the patient (including the MMSE and Montreal Cognitive Assessment) and a physical and neurological examination. All patients had routine laboratory assessment for causes of dementia, including blood work and brain imaging according to Canadian Consensus Guidelines (Gauthier et al., 2012). Imaging excluded significant vascular pathology; however, cerebrospinal or other amyloid biomarkers were not available. AD patients did not have vascular dementia based on a modified ischemic score >4. Medical comorbidity was recorded using the modified Cumulative Illness Rating Scale.

### Salivary Samples

Salivary samples were collected and prepared according to the manufacturer’s protocol. Participants were instructed not to eat one hour before testing and light washing was permitted prior to saliva collection. One saliva sample was collected per participant. The time of day for saliva collection varied throughout across participants. At a regular point in the data collection for each participant, the saliva collection task was announced, instructions were delivered, and the device was displayed and described. As the overall procedure was not time-limited, there was sufficient time for full samples from all individuals. We used the Oragene^®^ • DNA Self-Collection Kit OG-500 (DNA Genotek Inc., Ottawa, ON, Canada). Whole saliva was collected, placed inside the kit, and shaken. The kit contained an Oragene DNA-preserving solution. The ingredients of Oragene solution include ethyl alcohol (>24%) and Tris-HCl buffer (pH 8). As provided by established procedures, samples stored at room temperature were analyzed for DNA extraction, genotyping and the metabolomics analyses. Our previous pilot study included an analysis of five different saliva samples from CN adults collected at varying times of the day and stored at different temperatures to examine performance of the metabolomics profiling method. We observed that metabolites detected for each individual sample significantly discriminated the individuals despite small metabolite variations that may have been present for samples collected at different times of the day. In addition, there were relatively minor metabolite variations across a range of storage temperatures (room temperature, −20°C, −80°C; Zheng et al., 2012). All saliva samples were then preserved in −80°C for long-term storage and follow-up studies.

### Alzheimer’s Predictors From Six Risk Domains

In this section, we describe the procedures for obtaining the risk and biomarker data. These included two AD biomarker clusters using salivary samples: (1) salivary metabolites; and (2) genetic polymorphisms. The remaining domains were: (3) functional health; (4) lifestyle activity; (5) cognition; and (6) bio-demographic. We recruited diagnosed AD patients with mild form of dementia and limited available time for the testing session than the other two groups. Thus, we reduced the cognitive and physical load of our testing sessions for them. The total number of predictors differed between the clinical status discrimination analyses because the AD group was not tested on PP, BMI and lifestyle activities.

#### Metabolomics Procedure and Metabolite Panel Development

The metabolomics analyses leading to the present biomarker panels were performed in a previous study (Huan et al., 2018). In the study establishing the present biomarker panels, we applied a salivary metabolomics workflow with a differential chemical isotope labeling based liquid chromatography-mass spectrometry (LC-MS) platform using dansylation derivatization for an in-depth profiling of the amine/phenol submetabolome (Huan et al., 2018). This was adapted and extended from earlier pilot work on saliva metabolome profiling (Zheng et al., 2012). Five microliters saliva sample was aliquoted out from each individual sample and labeled with ^12^C-DnsCl. A pooled sample was prepared by mixing small aliquots of individual samples and then labeled with ^13^C-DnsCl. The ^12^C-labeled individual sample was then mixed with ^13^C-labeled pooled sample in a 1:1 amount ratio after the total concentration of the labeled metabolites was determined by LC-ultraviolet. The ^12^C-/^13^C- ion pairs belonging to the labeled amine/phenol submetabolome were extracted from raw LC-MS data by a peak pair picking program, IsoMS (Zhou et al., 2014). Missing values in the ion pair list was retrieved using Zero-fill (Huan and Li, 2015a) by searching and filling in the missing values from the raw MS data. Accurate intensity ratios of the ion pairs were reconstructed by their chromatographic peak ratios using IsoMS-Quant (Huan and Li, 2015b). After the LC-MS data processing, multivariate statistical analysis of the LC-MS data was conducted using SIMCA-P + 12.0 (Umetrics, Umeå, Sweden).

The metabolite biomarker panels were determined as follows (Huan et al., 2018). Pairwise statistical comparisons used orthogonal partial least squares-discriminant analysis (OPLS-DA) and volcano plot analyses. The diagnostic power of the common metabolites that were highly ranked with both statistical tools was then evaluated by receiver operating characteristic (ROC) analysis and linear SVM model using MetaboAnalyst (Xia et al., 2012). For positive or definitive metabolite identification, the peak pairs were matched against a Dns-standards library (Huan et al., 2015) by retention time and accurate mass. In addition, putative metabolite identification was performed based on accurate mass match of the peak pairs found to the metabolites in the Human Metabolome Database (Wishart et al., 2013) and the Evidence-based Metabolome Library using MyCompoundID (Li et al., 2013), with a mass tolerance of 5 ppm.

As a final step in the discovery phase, a machine learning linear SVM tool in MetaboAnalyst (Xia et al., 2012) was used to develop a diagnostic model for each of the three pairwise comparisons with: (1) 63 metabolites discriminating AD vs. CN; (2) 47 metabolites discriminating AD vs. MCI; and (3) two metabolites discriminating MCI vs. CN. In a follow-up validation phase, the diagnostic performance was further evaluated in a small (*n* = 27) but independent data set drawn from the same population. Specifically, validation was tested with similarly classified or diagnosed CN (age 68–75 years, 50% female), MCI; age 67–75 years, 50% female), and AD; age 53–91 years, 71.4% female) groups (Huan et al., 2018). The final diagnostic model best discriminated: (a) AD from CN with the AD metabolite panel (Methylguanosine, Histidinyl-Phenylalanine, Choline-cytidine); (b) AD from MCI with the AD/MCI metabolite (Amino-dihydroxybenzene, Glucosylgalactosyl hydroxylysine − H_2_O, Aminobutyric acid + H_2_); and (c) MCI from CN with the MCI metabolite panel (Glucosylgalactosyl hydroxylysine − H_2_O, Glutamine-carinitine; Huan et al., 2018). The additive score is comprised of the sum of all the values for each metabolite in the three diagnostic models and was used as the final metabolite panel in the present clinical status prediction analyses. Higher score indicated higher metabolite concentration in the diseased group.

#### Genetic Markers

DNA was manually extracted from 0.8 ml of saliva sample mix using the manufacturer’s protocol with adjusted reagent volumes. Genotyping was carried out by using a PCR-RFLP strategy to analyze the allele status for *APOE* (rs7412, rs429358), *CR1* (rs6656401), *CLU* (rs11136000) and *PICALM* (rs3851179). Genotyping was successful for the targeted SNPs for all present participants (McFall et al., 2015a). We included all three allelic combinations coded from 1 (lowest risk) to 3 (highest risk) for *CR1* (G/G = 1, G/A = 2, A/A = 3), CLU (T/T = 1, T/C = 2, C/C = 3), and *PICALM* (C/C = 1, C/T = 2, T/T = 3). For *APOE*, the study sample did not include any ε2/ε4 carriers and, therefore, the remaining five allelic combinations were coded from 1 (lowest risk) to 5 (highest risk; ε2/ε2 = 1, ε2/ε3 = 2, ε3/ε3 = 3, ε3/ε4 = 4, ε4/ε4 = 5).

#### Functional Health

In this category we included PP, BMI and gait (timed walk) as predictors. PP was calculated with systolic minus diastolic blood pressure (McFall et al., 2016). BMI was obtained from measurements of weight in kilograms and height in centimeters. BMI was calculated by taking the weight in kilograms divided by the square of one’s height in meters (kg/m^2^; MacDonald et al., 2011; Besser et al., 2014). A timed walking test was used to measure gait speed for all participants. The CN and MCI groups began the walking task from a standing position, a standard procedure for individuals with no mobility or dementia concerns. Specifically, the CN and MCI groups were measured by asking participants to walk a distance of 3 m, turn around, and walk back (MacDonald et al., 2017). AD patients began the task from a sitting position in an arm chair (i.e., the Timed Up and Go Test, a standard task in dementia research). Participants were seated in an armchair, and asked to get up and walk 3 m, turn around, walk and sit back in the chair. The time taken to complete this task was measured with a stopwatch in seconds. PP, BMI and timed walk were included as continuous variables.

#### Lifestyle Activity

The commonly used VLS Activity Lifestyle Questionnaire has 67 items measuring seven types of lifestyle engagement (Hultsch et al., 1999; Dolcos et al., 2012; Small et al., 2012) From the full inventory, we extracted items (*n* = 50) associated with the key dementia-related lifestyle aspects (cognitive, physical, and social). We evaluated two types of cognitive activities: (1) integrative information processing measured (*n* = 12) such as playing a musical instrument or household repairs, and (2) novel information processing (*n* = 27) such as completing jigsaw puzzles or reading the newspaper (Runge et al., 2014; Sapkota et al., 2017). Physical activity (*n* = 4) included jogging or gardening (Thibeau et al., 2017). Social activity (*n* = 7) included volunteering or visiting friends (Brown et al., 2016). The frequency of participation is rated on a 9-point scale with never (0), less than once a year (1), about once a year (2), 2 or 3 times a year (3), about once a month (4), 2 or 3 times a month (5), about once a week (6), 2 or 3 times a week (7), and daily (8). All the items were summed for each domain with higher scores representing greater frequency of activity (e.g., Small et al., 2012; Runge et al., 2014; Sapkota et al., 2017; Thibeau et al., 2017).

#### Cognition

The cognitive performance domain was represented by four standardized tests covering key aspects of performance known to be associated with differential normal and impaired aging, as well as dementia. First, to represent memory we used the standard VLS Word Recall task (Dixon and de Frias, 2004). From a pool of six equivalent lists, two different but comparable lists of 30 English words (i.e., six taxonomic categories with five words each) were used. Participants were given 2 min to study the list and 5 min to write down their answers. The total numbers of words correctly recalled from each list was averaged and used as the final score (Josefsson et al., 2012). Second, to measure EF (inhibition) we used the Stroop test (Scarpina and Tagini, 2017). This test consists of the standard three parts (Parts A, B and C), with the measures based on latencies. The score is the standardized Stroop interference index ([Part C− Part A]/ Part A), with a lower index reflecting better performance (MacLeod, 1991; de Frias et al., 2009; Diamond, 2013). Third, to measure speed we used the SRT task (Dixon et al., 2007). In this computer-based nonverbal response time task participants press a key on the response console with the index finger of their dominant hand at every occurrence of the target stimulus as quickly as possible. Response latencies were recorded to a precision of ±1 ms as the final score (McFall et al., 2015a). Fourth, for global cognition, we used examined the MMSE (Folstein et al., 1975), which measures performance on a scale of 0–30.

#### Bio-Demographic

The VLS personal data inventory was used to determine type and level of demographic risk (Anstey et al., 2013a; Sachdev et al., 2016). We examined education (total number of school years; Amieva et al., 2014; Cadar et al., 2016), age (in years; Small et al., 2011; Papenberg et al., 2015), and sex (male vs. female; Altmann et al., 2014; Li and Singh, 2014; McDermott et al., 2017).

### Statistical Analyses

RFA is a machine learning technology that applies a nonparametric approach to assess a large number of predictors in both complex and small data sets (Strobl et al., 2009; Kuhn and Johnson, 2013). These applications include biomarker predictions related to AD (Kaup et al., 2015; McDermott et al., 2017). We used RFA from the Party package (Hothorn et al., 2005) in RStudio version 1.0.136 (2017). The analysis combines regression trees based on a random selection of participants and variables. The regression trees are all combined and then used to rank variables according to their importance in predicting an outcome. The RFA party package accounts for any potential correlated predictor variables (Strobl et al., 2009). Any missing values were imputed using the missForest package (Stekhoven and Bühlmann, 2012). All the forests in our analyses examined 5,000 trees and a random sample of 10 predictors was tested at each potential split. The analyses ranked relative predictive importance based on standard statistical operations and procedural recommendations (Strobl et al., 2009). The metric for these rankings is termed “variable importance,” which specifies how important each factor is in discriminating two groups relative to all other factors in the model. Goodness of model fit for each RFA analyses was examined with area under the ROC curve (C-statistics). The C-statistic ranges from 0.50 to 1.00 can be interpreted as equivalent to the Area under the Curve in a ROC analysis where higher values are associated with better predictive models. Any variables with negative, zero, or small positive values are determined as not important predictors; these are represented to the left of the vertical dashed line. Variables right of the vertical dashed line with high positive values are considered to be important predictors (Strobl et al., 2009).

RFA was used to determine the most important predictors for discrimination in three pairwise groups (AD vs. MCI, AD vs. CN, MCI vs. CN). First, we tested which of the 13 risk factors were the most important predictors for discriminating clinical status for: (1) AD vs. CN and (2) AD vs. MCI. Second, we tested which of the 19 risk factors were the most important predictors for discriminating clinical status for (3) MCI vs. CN.

## Results

**Figure 1:**
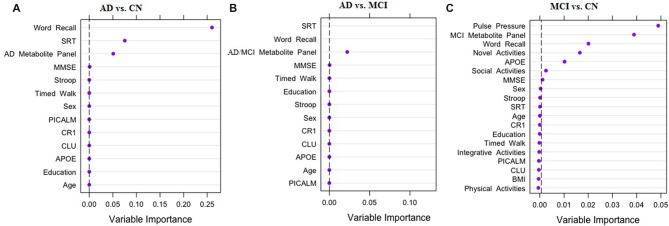
Results of random forest analyses (RFA) for three pairwise comparisons. The three panels of the figure display strongest predictors for discriminating clinical status: **(A)** Alzheimer’s disease (AD) vs. Cognitively Normal (CN); **(B)** AD vs. Mild Cognitive Impairment (MCI); **(C)** MCI vs. CN. Dashed black line is the cut off for variable importance in discriminating clinical status relative to other factors in the model. *APOE, Apolipoprotein E* (rs7412, rs429358); *CR1, Complement receptor 1* (rs6656401); *CLU, Clusterin* (rs11136000); *PICALM, Phosphatidylinositol-binding clathrin assembly protein* (rs3851179); BMI, Body Mass Index; SRT, Simple Reaction Time; MMSE, Mini-Mental State Exam.

Across the three multi-modal prediction analyses, we observed significant discrimination for the pairwise comparisons of the three clinical groups with predictors from the six AD biomarker risk domains (see Figure 1). First, for the AD vs. CN analysis, three important discriminative predictors were identified (C-statistic: 1.00). As shown in Figure 1A, the top predictors included two cognitive measures (speed and memory) and the AD metabolite panel. Specifically: (1) poorer memory performance; (2) slower speed performance; and (3) higher levels (greater risk) of the AD metabolite panel discriminated AD from CN group at a high level of importance. Second, for the AD vs. MCI analysis the same two cognitive predictors and the AD/MCI metabolite panel were identified as important predictors (C-statistic: 0.99). As can be seen in Figure 1B, A different order of importance was observed: (1) slower speed performance; (2) poorer memory performance; and (3) higher levels (greater risk) of the AD/MCI metabolite panel were the most important factors discriminating the AD and MCI groups. Third, as shown in Figure 1C, in the MCI vs. CN analysis, seven of the 19 predictors were identified as important in discriminating the groups (C-statistic = 0.94). Notably, the seven predictors represented five (of the six) risk domains (see Figure 1C). Specifically, the most important predictors for discriminating MCI from CN were: (1) higher PP; (2) higher levels of the MCI metabolite panel; (3) poorer memory performance; (4) lower frequency of novel cognitive activity; (5) elevated *APOE* risk; (6) decreased social activity; and (7) lower MMSE score.

## Discussion

We examined and compared neurodegenerative disease status predictions by selected modifiable and non-modifiable AD risk factors representing six prominent modalities. The relative prediction patterns were examined for the pairwise discrimination of the three groups (CN, MCI and AD). An important aim was to test the extent to which recently discovered salivary metabolomics biomarker panels (Huan et al., 2018) would perform in the competitive context of other biomarkers and risk factors of AD. Given the dynamic, insidious and multi-factorial nature of AD, it is likely that multiple modalities of risk biomarkers may contribute to the diagnosis of the disease. A corresponding emerging interest is in determining viable combinations of predictors for use in timely (early) detection and targeted (precise) intervention. Our results supported both the multi-modal predictor expectation and the potential valuable role that salivary-based biomarkers discovered through metabolomics analyses may play in identifying important components of AD biomarker batteries.

In our earlier metabolomics analyses, we detected salivary metabolite panels that were most accurate in discriminating the three groups (Huan et al., 2018). In this study, we examined how these panels performed in discriminating these groups in the competitive context of other known AD biomarkers or risk factors. In each of the three pairwise comparisons, the RFA results showed that the relevant metabolite panel was among the top important predictors. In fact, the AD and AD/MCI metabolite panels and the same two cognitive performance measures—i.e., speed and memory—in a different order discriminated AD from CN and AD from MCI. For the AD-CN comparison, the important predictors were memory, followed by speed and the AD metabolite panel. The AD metabolite panel represents pathways involved in AD protein regulation (Huan et al., 2018). For the AD-MCI comparison, speed and memory were among the most important predictors, and the associated AD/MCI metabolite panel also contributed at an important level. Dipeptides identified in both the AD metabolite panel and AD/MCI metabolite panel may reinforce the role of protein dysregulation in AD as a result of degraded proteins from amyloid or tau (Huan et al., 2018). Memory decline and impairment is a cardinal marker of preclinical dementia and is described as an oft-reported clinical symptom of aging and impairment (McKhann et al., 2011). Poorer memory performance in AD is consistent with key memory-related structural changes observed in the aging and impaired brain (Bartsch and Wulff, 2015). Specifically, hippocampal atrophy rates are comparatively greater in MCI than CN older adults and whole brain atrophy rates are greater in AD patients than MCI (Henneman et al., 2009). Slower speed performance has shown to be an early marker of lower and steeper cognitive decline (McFall et al., 2015a) and may be associated with poorer executive functioning and memory performance as well as increased dementia risk (Bäckman et al., 2005). Slower speed performance is also positively correlated with white matter tracts especially in the parietal and temporal cortices, and the left middle frontal gyrus (Turken et al., 2008).

Much attention in recent years has been on the detection of early signs—and their biomarker predictors—of transitions from CN to mildly impaired aging (Albert et al., 2011; Brainerd et al., 2013). Recently, this transition has also been investigated with unbiased metabolomics procedures (Zheng et al., 2012; Figueira et al., 2016; Liang et al., 2016; Huan et al., 2018). Two related challenges are that: (1) neither group is diagnosable with AD and (2) the exact probabilities of individual future conversion to AD are unknown. Moreover, both groups are likely to be in fluctuant, even overlapping, states of brain and cognitive aging—as indicated by the phenomenon of reversion (Manly et al., 2008; Koepsell and Monsell, 2012). Reviews of this challenge have led to the recommendation that multiple biomarkers and longitudinal data are advisable for differential classification. In the present study, these two groups were exactingly and objectively classified based on longitudinal data. Specifically, both groups were comprised of participants who were independently classified in status on two separate waves (about 4 years apart), underscoring the validity of the CN classification and the chronicity of the cognitively impaired classification. Our results reflect the challenge and relevance of considering multiple modalities of risk, and the apparent validity of carefully characterized groups. The RFA results showed that seven factors (representing five modalities) were found to be important predictors of impairment. The important predictors in order of significance were PP, MCI metabolite panel, memory, novel activities, *APOE*, social activities and MMSE. Elevated PP has been linked to cognitive impairment in MCI potentially in association with large artery stiffness (Yaneva-Sirakova et al., 2012; McFall et al., 2016). Our previously discovered MCI metabolite panel was the second important predictor of MCI status. This panel could be used in future targeted studies focusing on the differences and early markers of early memory impairment, as distinguished from normal memory decline. Notably, we employed a broad performance-based classification scheme that complements the standard clinical approach to MCI classification (Petersen et al., 2014). Two aspects of lifestyle activities—specifically, lower frequency of novel cognitive activities and decreased social engagement—predicted MCI group membership, in the context of the CN benchmark. The discriminative associations for these markers were in the expected direction, indicating that poorer lifestyle activities predicted probability of cognitive impairment (Verghese et al., 2006; Hughes et al., 2013). As previously reported in the literature (Brainerd et al., 2011; Dixon et al., 2014), AD genetic risk, as represented by *APOE* ε4+ genotypes predicted membership in the cognitive impairment group, in the context of the CN benchmark. Finally, poorer global cognition in the MCI group, an indication of future risk of dementia (O’Bryant et al., 2008), suggests that the MCI group maybe on an accelerated path to dementia onset compared to the CN group.

Overall, the results are consistent with the general perspective that risk markers from multiple modalities contribute to the prediction, classification or diagnosis of cognitive statuses such as MCI and AD. The results are also consistent with our expectation that new metabolite panels, derived from salivary metabolomics analyses, can be confirmed as among the better predictors of clinical status—but not the only predictor, especially for the crucial discrimination of CN and the impairment group. Along with notable strengths, we acknowledge several limitations. First, as a function of leveraging our earlier metabolomics study, our present sample sizes are relatively small. Although not perfect, this fact is statistically accommodated in the machine learning prediction analyses we used. Specifically, RFA are well suited to deal with small sample sizes because a large number of trees can be used in RFA models (Strobl et al., 2009). Larger number of trees allows for a large variety of predictor variable combinations to account for small sample sizes. Moreover, RFA outperforms other non-machine learning techniques (i.e., regression, and factor analysis) in that it accommodates: (1) small samples size in highly complex datasets (Maroco et al., 2011); (2) highly correlated datasets; and (3) large number of regression trees with specified set of predictors. The latter compensates for power issues as frequently observed in other statistical models with small sample sizes. We take the average of all 5,000 trees to employ a bagged variable importance measure—a procedure that leads to more stability and reduces the risk of over-fitting of the data. Nevertheless, some over-fitting of prediction models may occur, so further validation research is recommended. We specifically recommend follow-up validation studies, appropriate statistical evaluation, and larger sample sizes. Second, we deliberately incorporated a large number of predictors from multiple modalities—and all are established in a variety of independent research projects—but not all possible and potentially relevant predictors were included. For example, future research should include other standard AD biomarkers, such as cerebrospinal fluid β-amyloid (1–42), total tau, and phospho-tau-181 (Humpel, 2011), as well as other AD-specific neuroimaging biomarkers (e.g., hippocampal volume). Third, in order to more broadly generalize our results, we recommend studies recruit samples that are more demographically diverse, include alternative biospecimens or validate with autopsy confirmed AD cases. As noted, the metabolite panels used here were established in previous metabolomics research using the same groups. Future validation work would also benefit from targeting and testing these panels in different populations.

We tested a multi-modal array of risk biomarkers for their relative predictive power in discriminating three clinical status groups. This provides empirical evidence confirming the view that such multi-modal approaches can be valuable in research on neurodegenerative disease. In addition, the results also confirm that metabolomics procedures can produce biomarker panels that have relevance in the competitive context of other known risk factors for AD. Future work should examine such novel metabolite panels in the context of additional AD biomarkers. The results also show that modifiable risk factors can be important predictors of clinical status, even in the context of biomarkers from metabolomics and genomic approaches. At present, they appear to be especially relevant for the crucial discrimination of normal and impaired groups. The overall results lead to indications of potential use for validation and translation of non-invasive metabolite panels in pertinent combinations with established multi-modal biomarkers for early dementia risk detection and intervention programs. Early and precise risk detection can lead to personalized risk management and other intervention strategies for older adults at elevated risk for AD (Barnes and Yaffe, 2011; Anstey et al., 2014; Olanrewaju et al., 2015).

## Data Availability

Datasets are available upon request from the corresponding authors.

## Author Contributions

RD is the director of the VLS and was responsible for all data and sample collection. RD and SS designed and planned the present research and statistical analyses. SS and TH assembled and validated the data set. LL is the director of the metabolomics lab and led TH, TT and JZ in performing the metabolomics analyses that contributed the metabolite biomarker panels. SS performed the data assembly and the present statistical analyses, with contributions from RD and TH. SS wrote the drafts of the article, with assistance from RD, LL, RC and TH. RC performed the clinical diagnoses of AD and consulted on the MCI classifications.

## Conflict of Interest Statement

The authors declare that the research was conducted in the absence of any commercial or financial relationships that could be construed as a potential conflict of interest. The reviewer CJ and handling Editor declared their shared affiliation at the time of the review.

## Footnotes

^1^http://www.hmdb.ca/metabolites/HMDB0000585

^2^http://www.hmdb.ca/metabolites/HMDB0000062
